# A Portable, Wireless Photoplethysomography Sensor for Assessing Health of Arteriovenous Fistula Using Class-Weighted Support Vector Machine

**DOI:** 10.3390/s18113854

**Published:** 2018-11-09

**Authors:** Paul C.-P. Chao, Pei-Yu Chiang, Yung-Hua Kao, Tse-Yi Tu, Chih-Yu Yang, Der-Cherng Tarng, Chin-Long Wey

**Affiliations:** 1 Institute of Electrical and Control Engineering, National Chiao Tung University, Hsinchu 300, Taiwan; mekool.eed00@g2.nctu.edu.tw (P.-Y.C.); yunghwa.kao@gmail.com (Y.-H.K.); tu.tseyi@gmail.com (T.-Y.T.); clwey@cc.nctu.edu.tw (C.-L.W.); 2 Division of Nephrology in Taipei Veterans General Hospital, Taipei 112, Taiwan; cyyang3@vghtpe.gov.tw (C.-Y.Y.); dctarng@vghtpe.gov.tw (D.-C.T.)

**Keywords:** photoplethysmography (PPG) sensor, support vector machine (SVM), arteriovenous fistula (AVF)

## Abstract

A portable, wireless photoplethysomography (PPG) sensor for assessing arteriovenous fistula (AVF) by using class-weighted support vector machines (SVM) was presented in this study. Nowadays, in hospital, AVF are assessed by ultrasound Doppler machines, which are bulky, expensive, complicated-to-operate, and time-consuming. In this study, new PPG sensors were proposed and developed successfully to provide portable and inexpensive solutions for AVF assessments. To develop the sensor, at first, by combining the dimensionless number analysis and the optical Beer Lambert’s law, five input features were derived for the SVM classifier. In the next step, to increase the signal-noise ratio (SNR) of PPG signals, the front-end readout circuitries were designed to fully use the dynamic range of analog-digital converter (ADC) by controlling the circuitries gain and the light intensity of light emitted diode (LED). Digital signal processing algorithms were proposed next to check and fix signal anomalies. Finally, the class-weighted SVM classifiers employed five different kernel functions to assess AVF quality. The assessment results were provided to doctors for diagonosis and detemining ensuing proper treatments. The experimental results showed that the proposed PPG sensors successfully achieved an accuracy of 89.11% in assessing health of AVF and with a type II error of only 9.59%.

## 1. Introduction

Arteriovenous fistulas (AVF) are the lifelines of the end stage renal disease (ESRD) patients. AVF refers to the surgical connection between the brachial artery and vein for hemodialysis (HD). Having built AVF by surgery, the brachial vein is gradually arterialized, and then its vessel wall eventually becomes strong enough to sustain repeated needle-insertions during HD treatments. However, after a long term of HD treatments, patients may suffer from various AVF dysfunctions, which result in low blood flow volume (BFV) or narrowed vessels, such as stenosis, calcification, thrombosis, etc. According to the National Kidney Foundation [1], the health of AVF is assessed by measuring the BFV flowing insides, which should be at least 600 mL/min for performing HD treatments. In hospital, BFV at AVF is often assessed by using an ultrasound Doppler machine, which is expensive, bulky, and hard-to-use. Therefore, an alternative solution is proposed herein to provide ESRD patients with a portable, reliable, and inexpensive way to assess their AVF at home. 

There are published works about assessing AVF using small-sized sensors. Yeih D. F. et al. [2] published research about assessing AVF using the stethoscope auscultation methods by applying support vector machine (SVM) algorithms. The experimental results reveal an accuracy of 84.3%. Similarly, Wang H. Y. et al. [3] presented a small-sized stethoscope sensor for detecting the stenosis of AVF, which shows 87.84% accuracy with a very large database. However, the unavoidable environmental sound is a great issue for the sensitive stethoscope sensors [4]. Du Y.-C. et al. [5] presented a bilateral photoplethysmography (PPG) sensor system, assessing AVF using a neural network algorithm with very high accuracy of 94.82%, but with only eleven subjects. Chiang P. Y. et al. [6] published their study on monitoring the BFV at AVF using a single PPG sensor, and the clinical results showed correlation of 0.7176. However, very serious overestimation occurred in Reference [6], which resulted in a very high (>50%) type II error. To the best of the authors’ knowledge, there is still no portable, small-sized PPG sensor providing a reliable and inexpensive solution to AVF assessment. To make advances in sensor accuracy and functionality, this study was intended to design a portable, wireless PPG sensor for assessing AVF by using class-weighted SVM.

Nowadays, PPG sensors are widely used in various physiological applications, such as monitoring heart rate [7,8,9], heart rate variability [10,11,12], blood pressure [13,14,15], and blood oxygen level [16,17,18] etc. Moreover, since the hardware of PPG sensors can be manufactured to small sizes with low cost, PPG sensors can be implemented into various wearable applications, such as bracelets [19], watches [20], patches [21], earpieces [22], rings [23], etc. Despite many advantages, the ambient light interferences and the motion artifacts are the two great challenges for PPG sensors. On one hand, to cancel the ambient light interferences, Kim J. et al. [24] published a readout circuit for cancelling ambient light from sensors. Additionally, Chiang P.Y. et al. [25] presented a light compensation circuit for compensating the signal offset caused by environmental light. On the other hand, to deal with the motion artifacts, Zhang Z. et al. [26] presented a new algorithm to estimate the heart rate from a wrist PPG seriously polluted by the motion artifacts. Dao D. et al. [27] published an algorithm for detecting the motion artifacts and for evaluating the usability of PPG signals. Wijshoff R.W.C.G.R. et al. [28] introduced a sensor fusion system combining a PPG, Electrocardiogram (ECG), and accelerometer to detect and compensate the motion artifacts using the frequency lock loop. Yang D. et al. [29] presented another research about removing the motion artifacts from PPG signals using adaptive spectrum noise cancellation with the aid of an accelerometer. In this work, the ambient light interferences were cancelled by using the low-pass filter in the readout circuitries and the normalization in the features extraction. Furthermore, a series of digital signal processing and algorithms of checking signal quality were also implemented herein to handle the issue of motion artifacts.

This work is organized as follows. In Section 2, dimensionless number analysis and the optical Beer Lambert’s law are introduced to derive the input features for SVM algorithm. Section 3 describes the system design, including the readout circuitries, the digital signal processing, and the assessing algorithms. In Section 4, the clinical validation and the experimental results are shown. Section 5 discusses the experimental results in comparison to other similar publications. Section 6 concludes the work. 

## 2. Theories and Principles 

Theories and principles of the proposed PPG sensors are described in this section to determine the exact input features for the SVM algorithm.

### 2.1. Dimensionless Analysis of Hemodynamic Model 

**Figure 1:**
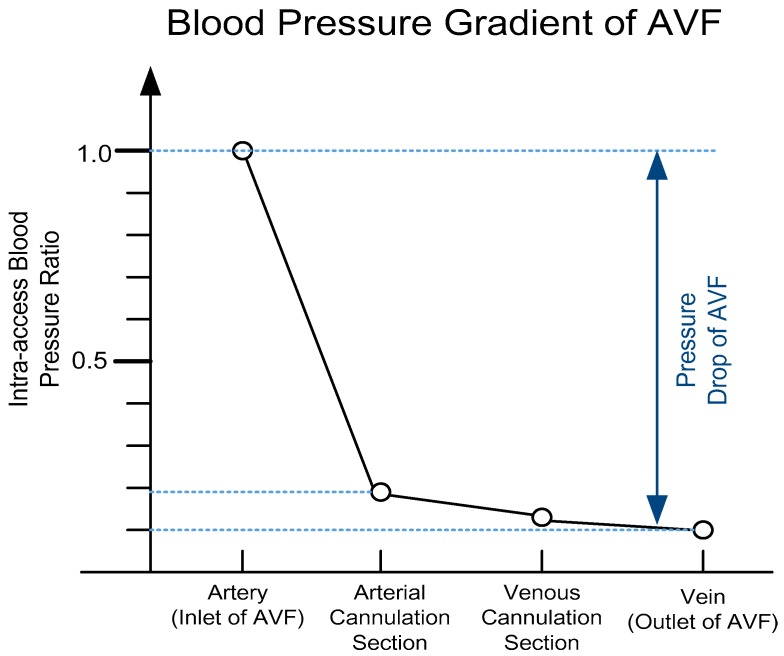
The blood pressure gradient of arteriovenous fistula (AVF), where it can be observed that there is a severe blood pressure drop (>90% decrease) across AVF (based on Reference [1]).

In this work, to describe the hemodynamic model of AVF, the dimensionless number analysis of the turbulence power loss model, where its parameters were proved to be complete, as described in Reference [30], is introduced herein as

(1) $$\frac{\varepsilon_{p}}{\rho \left(Q^{3}/r^{4}\right)}=f_{1}\left(Q_{\omega },S,\beta_{BC},Re,Ca,St\right)\;$$

where *ε_p_* denotes the average power dissipation rate of the blood flow; *ρ* denotes the blood density (about 1.056 g/cm^3^); *Q* and *Q*_ω_ denote the average BFV and its waveform shape; *r* denotes the radius of the measured vessel; *S* and *β_BC_* denote the geometry of blood vessel and the boundary condition respectively, which are typically viewed as constants; where the dimensionless parameters are

(2) $$Re=\frac{2\rho Q}{\pi r\eta }\;$$

(3) $$Ca=\frac{16r^{4}E}{\rho Q^{2}}\;$$

(4) $$St=\frac{8r^{3}f_{\mathrm{HR}}}{Q}\;$$

where *Re* denotes the Reynolds number; *η* denotes the dynamic viscosity of blood (about 0.035 g/cm∙s); *Ca* denotes the Cauchy number; *E* denotes the Young’s elasticity modulus of the vessel wall, which is assumed to be a constant; *St* denotes the Strouhal number; *f*_HR_ denotes the heart rate frequency. In Equation (1), the average power dissipation rate *ε_p_* can be defined as the difference between the total energy dissipation rate and the energy dissipation rate of the steady part of flow as

(5) $$\begin{array}{ll} \varepsilon_{p} & =\varepsilon_{t}-\varepsilon_{s}\\ & =\frac{1}{T}\oint p(t)q(t)dt-\frac{1}{T^{2}}\oint p(t)dt\oint q(t)dt\; \end{array}$$

where *ε_t_* and *ε_s_* denote the average of total power dissipation and the average of the steady component of flow; *T* denotes the heart rate period, which is 1/*f*_HR_; *p*(*t*) and *q*(*t*) denote the instantaneous blood pressure and BFV, respectively. Moreover, Equation (5) can be further simplified since the blood pressure at the outlet of AVF is very small and can be neglected, as shown in Figure 1. 

Therefore, in Equation (5), the energy power dissipation of AVF can be approximated using only the blood pressure at the inlet of AVF as

(6) $$\begin{array}{ll} \varepsilon_{p} & \equiv \frac{1}{T}\oint p_{in}(t)q_{in}(t)dt-\frac{1}{T^{2}}\oint p_{in}(t)dt\oint q_{in}(t)dt\\ & =\frac{1}{T}\oint PP_{\omega }QQ_{\omega }dt-\frac{1}{T^{2}}\oint PP_{\omega }dt\oint QQ_{\omega }dt\\ & =\frac{PQ}{T}\oint P_{\omega }Q_{\omega }dt-\frac{PQ}{T^{2}}\\ & =\frac{PQ}{T}k-\frac{PQ}{T^{2}}\; \end{array}$$

where *p_in_*(*t*) and *q_in_*(*t*) denote the blood pressure and BFV at the inlet of AVF; *P* and *P*_ω_ denote the average blood pressure and the corresponding waveform shape; *k* denotes the power loss ratio caused by waveform shape, which is assumed to be a constant. Furthermore, *P* can be expressed using the mean artery pressure, which is defined as in Reference [31]

(7) $$P\equiv \frac{1}{3}SBP+\frac{2}{3}DBP\;$$

where *SBP* and *DBP* denote systolic blood pressure (SBP) and diastolic blood pressure (DBP), respectively. Therefore, substituting Equation (7) into Equation (6) gives

(8) $$\varepsilon_{p}\equiv \left(\frac{1}{3}SBP+\frac{2}{3}DBP\right)\left(\frac{Q}{T}k-\frac{Q}{T^{2}}\right)\;$$

Therefore, from Equations (2)–(8), the average BFV in Equation (1) can be written as

(9) $$Q=f_{2}(SBP,DBP,f_{HR},r)\;$$

Note that the exact orders of each parameter remain unknown from dimensionless analysis. However, the SVM algorithm is still feasible by applying the derived parameters as the input features, and by selecting a proper kernel function. 

### 2.2. Optical Theory of PPG Sensors

**Figure 2:**
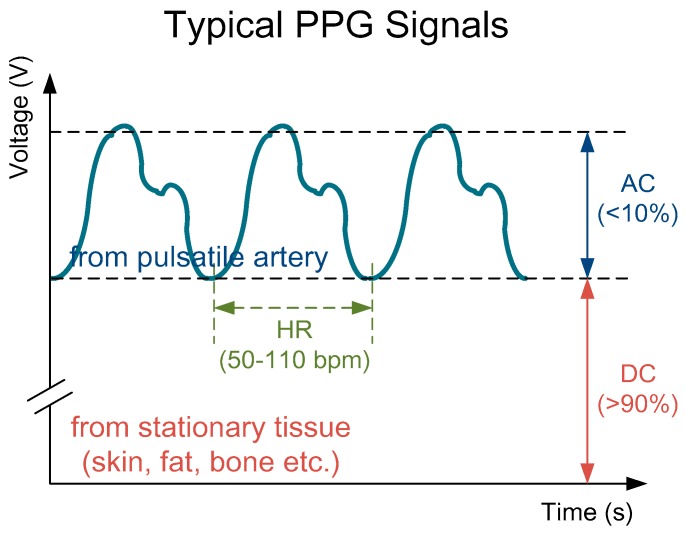
A typical photoplethysmography (PPG) signal consisting a large stationary part (>90%) and a small pulsating part (<10%) with a pulse frequency same as the heart rate (about 50–110 bpm).

PPG sensors are optical sensors for measuring intravascular blood volume changes noninvasively. A typical PPG signal is composed of a small pulsating part denoted as AC, and a large stationary part denoted as DC, as shown in Figure 2. 

The mathematical equation of PPG signals can be described using the well-known optical Beer Lambert’s law [32] as

(10) $$I_{PPG}=\left(I_{0}+I_{amb}\right)\cdot e^{-\varepsilon_{t}c_{t}s_{t}}\cdot e^{-\varepsilon_{b}c_{b}s_{b}}\;$$

where *I_PPG_* denotes the measured PPG signals; *I*_0_ and *I_amb_* denote the intensity of the incident light and the ambient light; *ε_b_* and *ε_t_* denote the light absorption coefficients of blood and tissues respectively, which are functions of light wavelength; *c_b_* and *c_t_* denote the Mohr concentration of blood and tissues, respectively; *s_b_* and *s_t_* denote the length of light transmission path in blood and tissues, respectively. Moreover, Equation (10) can be factored to DC and AC terms by applying the Taylor series approximation as

(11) $$\begin{array}{ll} I_{PPG} & \equiv \left(I_{0}+I_{amb}\right)\cdot \left(1-\varepsilon_{t}c_{t}s_{t}\right)\cdot \left(1-\varepsilon_{b}c_{b}s_{b}\right)\\ & =\left[\left(I_{0}+I_{amb}\right)\cdot \left(1-\varepsilon_{t}c_{t}s_{t}\right)\right]+\left[\left(I_{0}+I_{amb}\right)\cdot \left(1-\varepsilon_{t}c_{t}s_{t}\right)\cdot \left(-\varepsilon_{b}c_{b}s_{b}\right)\right]\\ & =DC+AC\;. \end{array}$$

Furthermore, to cancel the ambient light interferences, the perfusion index (PI) is introduced herein, as described in References [33,34], which is defined as AC divided by DC, as

(12) $$PI=\left|\frac{AC}{DC}\right|=\varepsilon_{b}c_{b}s_{b}\;$$

where an absolute symbol is added for calculating convenience. From Equation (12), it can be observed that the ambient light interferences (*I_amb_*) are normalized and will not affect later algorithms. Furthermore, considering the blood oxygen saturation levels, the term *ε_b_c_b_* in Equation (12) can be further expanded as in Reference [35]

(13) $$\varepsilon_{b}c_{b}=SpO_{2}\cdot \varepsilon_{HbO}+\left(1-SpO_{2}\right)\cdot \varepsilon_{Hb}\;$$

where *SpO_2_* denotes the blood oxygen saturation levels; *ε*_HbO_ and *ε*_Hb_ denote the light absorption coefficients of oxy-hemoglobin and hemoglobin, respectively. Moreover, the light transmission path in blood vessel (*s_b_*) can be viewed as the diameter of the measured vessel. Hence, combining Equations (12) and (13), the radius of the measured vessel can be written as

(14) $$r=\frac{s_{b}}{2}=\frac{1}{2}\frac{PI}{SpO_{2}\cdot \varepsilon_{HbO}+\left(1-SpO_{2}\right)\cdot \varepsilon_{Hb}}\;$$

Therefore, substitution of Equation (3) into Equation (9) yields

(15) $$Q=f_{2}(SBP,DBP,SpO_{2},f_{HR},PI)\;$$

In summary, the five input features for the SVM algorithm, including *SBP*, *DBP*, *SpO_2_*, *f_HR_* and *PI*, are derived by combining the dimensionless analysis and the optical theory of PPG signals. 

## 3. System Designs

**Figure 3:**
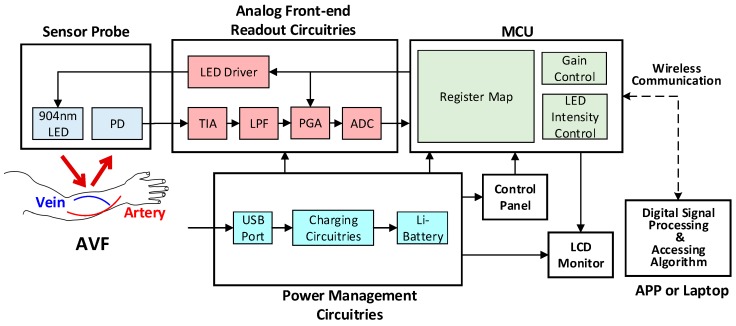
The system architecture of the proposed wireless PPG sensor system.

**Figure 4:**
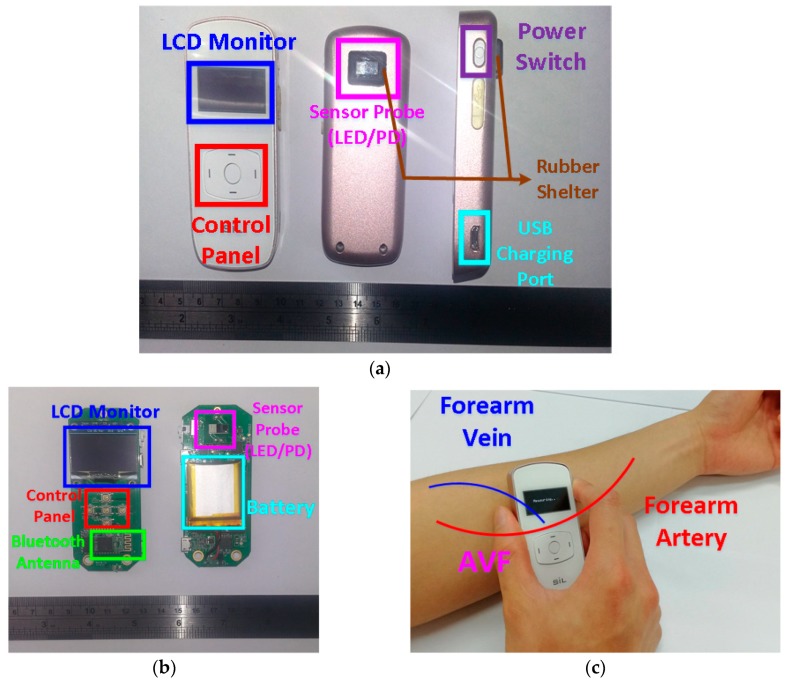
(**a**) The front, back, and side of the designed PPG sensor for assessing AVF. (**b**) The hardware circuitries of the designed PPG sensor. (**c**) The photo of the proposed PPG sensor measuring.

The architecture of the proposed PPG sensor system is shown in Figure 3, which is mainly composed of the sensor probe, the analog front-end readout circuitries, the microcontroller unit (MCU), and the software algorithms in APP or laptop. The photo of the proposed portable PPG sensor, which is only 9 cm × 3.5 cm × 1.5 cm in size, is shown in Figure 4a. Moreover, the sensor probe, the readout circuitries, the control panels, the Bluetooth antenna, the liquid crystal display (LCD) monitor, and the chargeable lithium battery are all implemented on a printed circuit board (PCB), as shown in Figure 4b. Moreover, the photo of the proposed sensor measuring is shown in Figure 4c, where the proposed PPG sensor can be operated by patients themselves without the aids of medical personnel. The detailed design of the hardware circuitries and the software algorithms are described as follows. 

### 3.1. Hardware Circuitries Design

**Figure 5:**
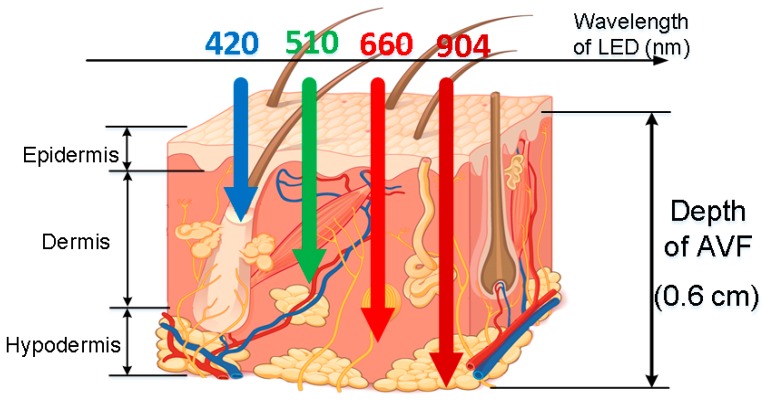
Generic light traces with different wavelengths, penetrating into skin tissues with different depth, where the typical depth of AVF (0.5–0.6 cm [1]) are corresponding to 904 nm wavelength (figure based on Reference [6]).

The analog readout circuitry of the proposed PPG sensors is composed of a light emitted diode (LED), a programmable LED driver, a photodiode (PD), a transimpedance amplifier (TIA), a low-pass filter (LPF), a programmable gain amplifier (PGA), and an analog-to-digital converter (ADC), as shown in Figure 3. First, at the probe, the LED with 904 nm wavelength is used, of which light may penetrate the typical depth of the AVF, as shown in Figure 5. Second, the TIA circuit is proposed to transform the current signals from the PD into voltage signals, which are much preferred in discrete circuit applications. Next on, to deal with the ambient light flickers from power lines (50 or 60 Hz), where the frequency is much higher than the heart rate frequency (0.8 to 2 Hz), the LPF with cutoff frequency 10 Hz is designed herein. Moreover, the PGA circuit and the programmable LED driver are designed herein to increase the signal-noise ratio (SNR), as well as to avoid the sensed signals from saturation by controlling the amplitudes of PPG signals to fully use the dynamic range of the ADC. Finally, the converted digital PPG signals from the ADC will be transmitted to the MCU and then transmitted to smart phone APPs or laptops via Bluetooth for further signal processing and assessing algorithms. 

### 3.2. Software Algorithm Design

#### 3.2.1. Digital Signal Processing

**Figure 6:**
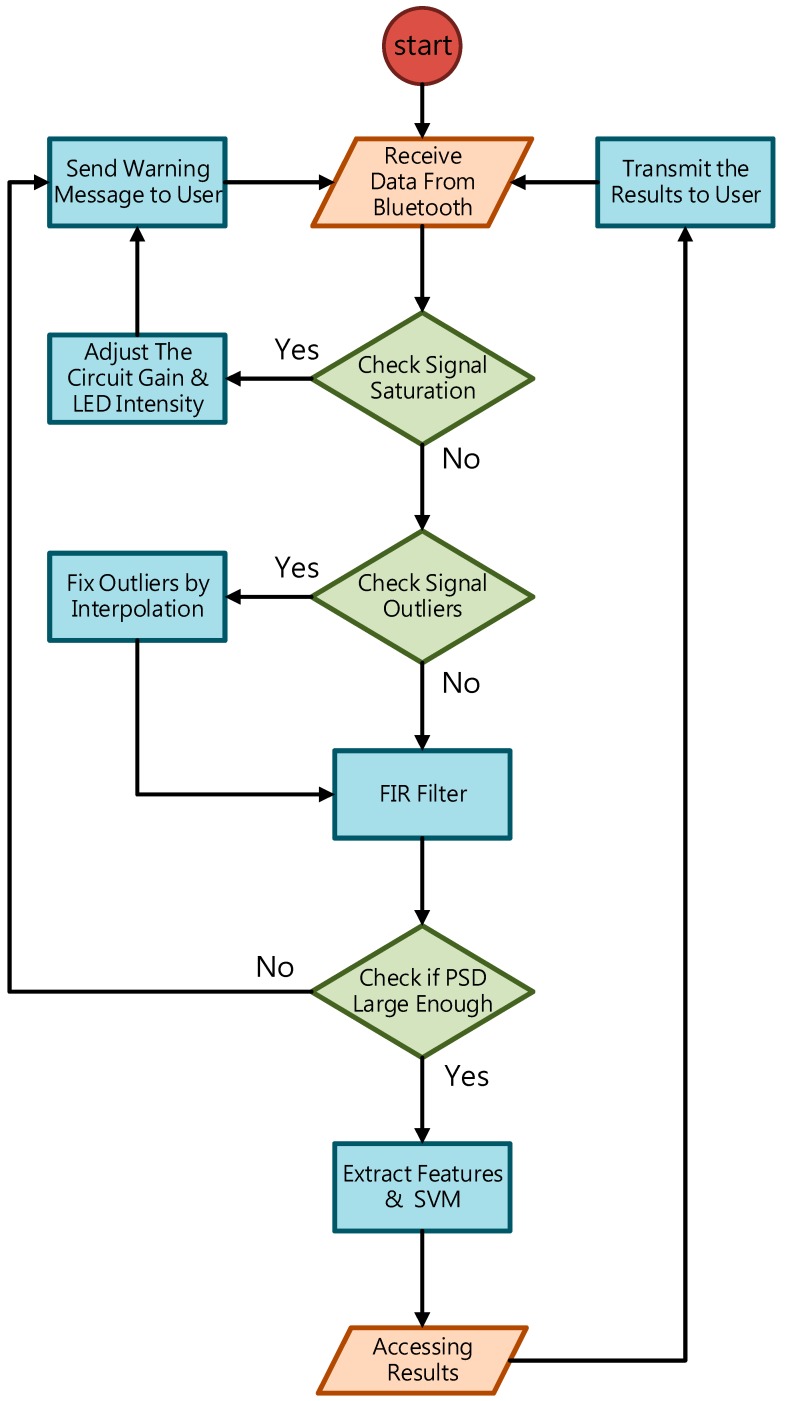
The flow chart of the digital signal processing in the proposed PPG sensor system.

**Figure 7:**
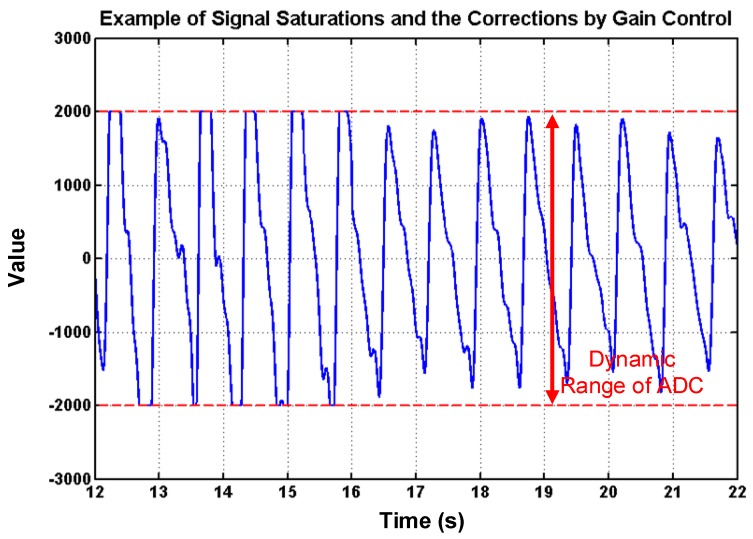
PPG signal saturations and the corrections by using gain control, where it can also be seen that the amplitudes are controlled to full dynamic range of analog-digital converter (ADC) to increase the signal-noise ratio (SNR).

**Figure 8:**
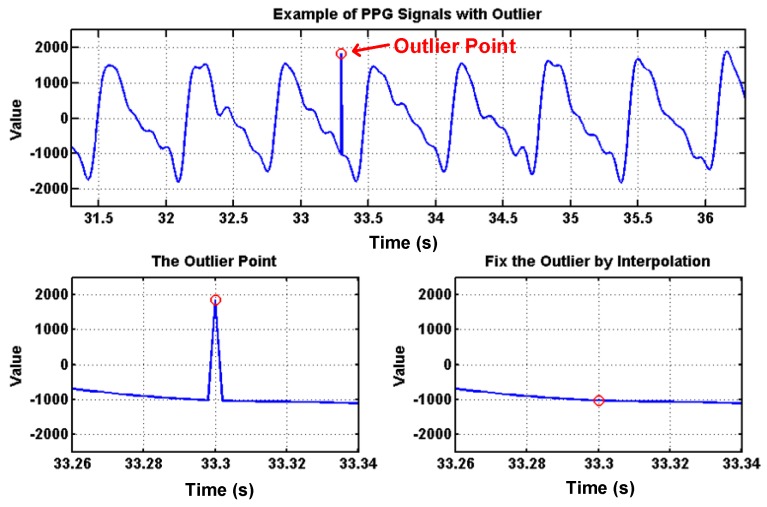
PPG signals with an outlier point, which can be detected by slope and can be fixed by using interpolation.

**Figure 9:**
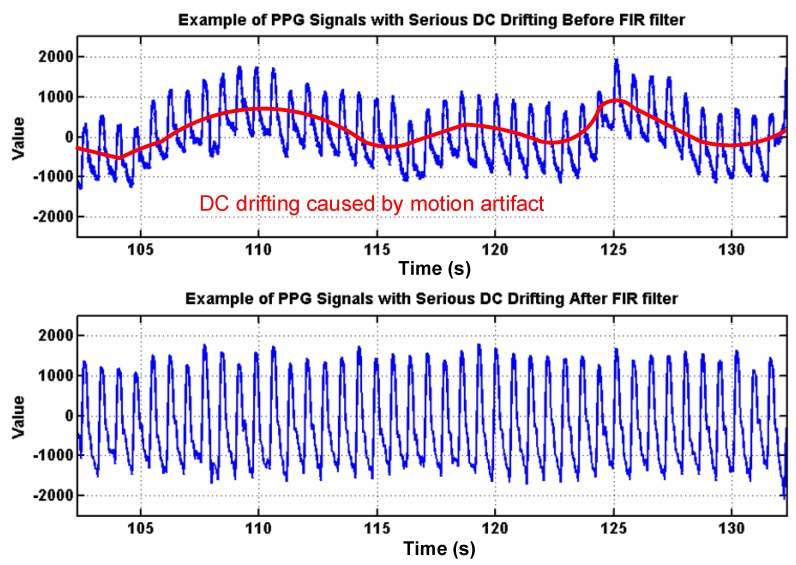
Effects of the proposed finite impulse response (FIR) filter to cancel the DC drifting caused by low-frequency motion artifacts, such as vasomotion, respiration.

**Figure 10:**
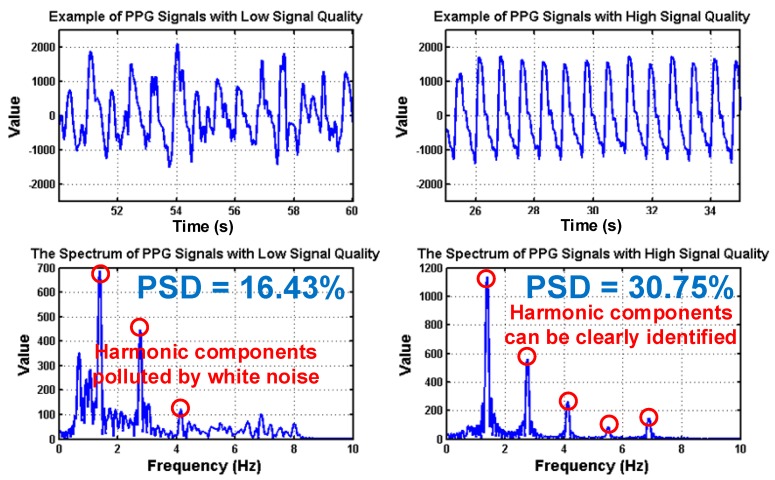
PPG signals with low and high signal quality following the corresponding spectrums, where the values of power spectrum density (PSD) are also shown.

To deal with PPG signal anomalies, four digital signal processing algorithms are designed herein, including the saturation detection, the outlier fixing, the finite impulse response (FIR) filter, and the power spectrum density (PSD) evaluation, as shown in Figure 6. First, the signal saturations are detected to avoid failure in extracting the features from PPG signals. The programmable LED light intensity driver and the PGA in the readout circuitries are designed to deal with signal saturations by decreasing the signal amplitude to fit the full dynamic range of ADC, as shown in Figure 7. Second, the signal outlier fixing process is introduced to fix the pulse noise caused by electrostatics due to constant contact of sensor and skins. The signal outlier points can be detected by observing the slope of PPG signals, and then they can be fixed by using interpolation, as shown in Figure 8. Next, to deal with the low frequency motion artifacts, such as vasomotion (0.8–0.25 Hz), respiration (0.2–0.4 Hz), etc., an FIR bandpass filter with cutoff frequencies 0.82 Hz and 10 Hz is designed to cancel the DC drifting, as shown in Figure 9. Finally, for other non-periodic motion artifacts, such as patients’ talking, moving, etc., the PSD evaluation method is introduced herein to provide a quantitative index for describing the quality of PPG signals [6]. It can be seen from Figure 10 that PPG signals are contaminated by motion artifacts with much lower PSD values due to the large white noise induced by the non-periodic motion. Therefore, by evaluating the value of PSD, proper warning messages can be sent to users to reduce the motion artifact noise. In summary, the four signal processing algorithms mentioned above are able to check and fix the signal anomalies in real time and to increase the SNR, as well as to reduce the motion artifacts. 

#### 3.2.2. Class-Weighted Support Vector Machine

In this work, a class-weighted SVM classifier ws proposed for assessing the health of AVF. SVM is a popular supervised learning model for data classification. Its basic concept is to construct an optimized hyper-plane from the mapping results of a kernel function to classify the data into different classes [36]. However, a traditional SVM model may sometimes produce sub-optimal results while classifying an imbalanced dataset, which is often the case in bio-medical applications. Therefore, to deal with imbalanced classifications, the class-weighted SVM classifier model was introduced herein, as described in Reference [37], where the objective function is to minimize

(16) $$\underset{\alpha }{\min}\left\{0.5\sum_{i}^{n}\sum_{j}^{n}\alpha_{i}\alpha_{j}y_{i}y_{j}G\left(x_{i},x_{j}\right)-\sum_{i}^{n}\alpha_{i}\right\}\;$$

subjected to

(17) $$\sum_{i}^{n}y_{i}\alpha_{i}=0\;$$

(18) $$0<\alpha_{i}{}^{+}<C^{+}\;$$

(19) $$0<\alpha_{i}{}^{-}<C^{-}\;$$

where *n* denotes the number of training samples; *α* denotes the Lagrange multiplier; *x* denotes the features vector with the class label *y*; *G* denotes the kernel function; *α^+^* and *α^−^* denote the Lagrange multiplier to positive and negative samples, respectively; *C*^+^ and *C*^−^ denote the misclassification costs for positive and negative samples, respectively. Additionally, it is reported that the class-weighted SVM classifier can be optimized by setting the ratio *C*^+^ / *C*^−^ to the ratio of the sample size of the two classes [38]. Moreover, from Equation (4), the five input features are determined for the classifier, as listed in Table 1. 

Note that all features in Table 1. are normalized to values between −1 and 1 before training to avoid weighted errors. Furthermore, since there is neither order information from Equation (4) nor enough statistical knowledge of features from subjects, five different kernel functions are proposed herein, including a radial basis function (RBF) kernel, linear kernel, second-order polynomial, third-order polynomial, and fourth-order polynomial, which are as in Reference [39]

(20) $$G_{RBF}(x_{i},x_{j})=e^{-{\left\|x_{i}-x_{j}\right\|}^{2}/2\sigma^{2}}\;$$

(21) $$G_{Linear}(x_{i},x_{j})=x_{i}\prime x_{j}\;$$

(22) $$G_{Poly,d}(x_{i},x_{j})={\left(x_{i}\prime x_{j}+1\right)}^{d}\;$$

where *G_RBF_* denotes the RBF kernel; *σ* denotes the scaling factor of RBF kernel; *G_Linear_* denotes the linear kernel; *G_Poly,d_* denotes the *d*-th order polynomial kernel; *d* is set as 2, 3, and 4 to employ the second-, third-, and fourth-order polynomial kernels, respectively. Note that the orders of the polynomial kernel higher than 4 is not used herein due to heavy computation loading. The values of *C^+^, C^−^* and *σ* are determined by using the grid search technique via the Matlab Machine Learning Toolbox, aiming at optimizing the accuracy of the SVM classifier. Furthermore, the *k*-fold cross validation process is introduced herein to validate the performance of classifiers. The steps of the *k*-fold cross validation process are as

Step (1) All subjects are randomly grouped into *k* non-overlapping subsets.
Step (2) One subset is tested with other *k*-1 subsets as the training set.
Step (3) Step (2) repeats *k* times with different subsets as testing.
Step (4) The average accuracy and error can be then calculated.

In this work, *k* is set to 10. 

## 4. Clinical Validation

### 4.1. Experimental Setup

According to the guideline from the National Kidney Foundation [1], BFV at healthy AVF should be at least 600 mL/min for performing HD treatments. Therefore, subjects can be grouped into the positive class (healthy AVF) and the negative class (dysfunctional AVF) by evaluating whether the BFV at AVF is larger than 600 mL/min. Prior to the experiments, the nephrologists label the subjects into the positive and the negative classes based on whether BFV exceeds 600 mL/min by the ultrasound Doppler machine. This labeling by the nephrologists serves as the ground truth for the SVM classifier. In this work, there were a total of 101 ESRD subjects with AVF participating in the experiments, including 73 subjects labeled as positive class and 28 subjects labeled as negative class. Before starting the measurements, all subjects are asked to rest for at least 15 min. During the experiments, subjects were asked to sit still and each of them was measured for 1 minute using the proposed PPG sensor. After that, the SBP and DBP values of each subject were measured by an electronic sphygmomanometer and the SpO_2_ value was measured using an oximeter. Moreover, the reproducibility of the proposed PPG sensor was also tested. There were 5 volunteers participating in the reproducibility experiment, including 3 patients with healthy AVF and 2 patients with dysfunctional AVF. Each of them was measured 3 times using the proposed PPG sensors to find out the reproducibility on a single patient. 

### 4.2. Experimental Results

**Figure 11:**
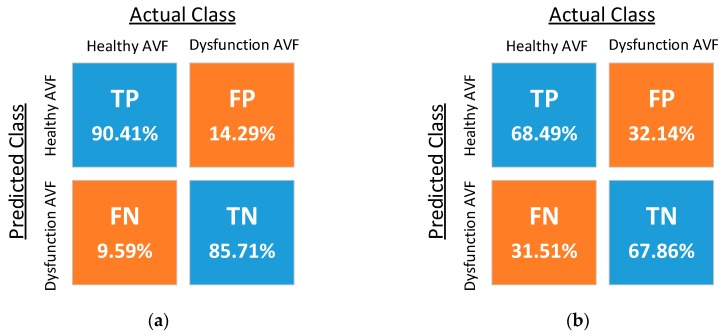

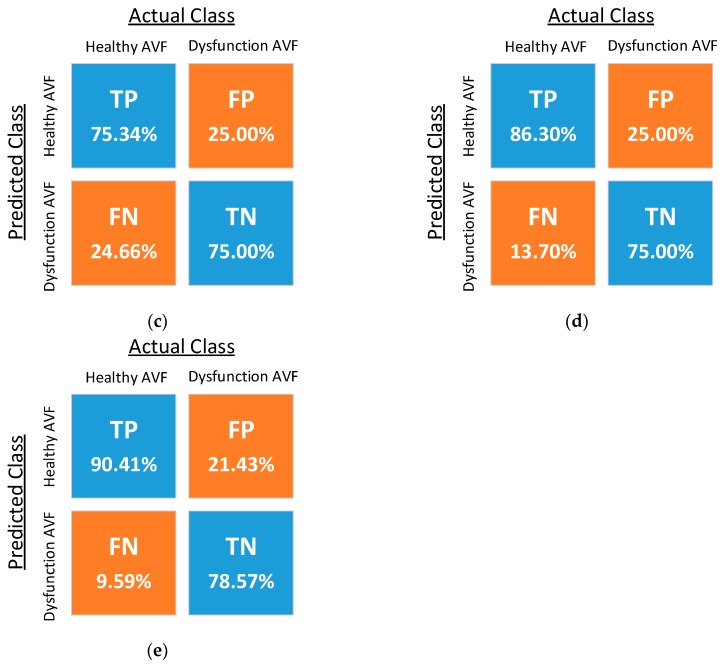
Confusion matrices of SVM classifiers with (**a**) the radial basis function (RBF) kernel, (**b**) the linear kernel, (**c**) the second-order polynomial kernel, (**d**) the third-order polynomial kernel, and (**e**) the fourth-order polynomial kernel.

The confusion matrices resulting from the classification using five different kernel functions are shown in Figure 11, where TP, FN, FP, and TN denote the ratios of the true-positive, false-negative, false-positive, and true-negative samples, respectively. The optimized costs, scaling factor, and performance of the five classifiers are shown in Table 2. The experimental results of the reproducibility test are shown in Table 3, where the proposed PPG sensors achieve high reproducibility for the SVM classifiers using the RBF kernel and fourth-order polynomial kernel. 

## 5. Discussion

Based on the experimental results in Table 2, the SVM classifier with RBF kernel achieves higher accuracy and lower error as compared to others. In many similar researches about SVM classification on bio-medical applications [40,41,42], the classifier with the RBF kernel often renders better results as opposed to the others with linear and polynomial kernels. The reason is that the features’ space induced by RBF is in an infinite dimension, whilst the others by linear or polynomial kernels are kept as in the original dimension [43]. However, despite the good results, the computation loadings of RBF kernels may increase significantly while training a large database. Moreover, the SVM classifier using RBF kernel is more likely to encounter over-fitting issues [44]. Therefore, the linear kernel, polynomial kernel, and other custom-made kernels are much preferred for linear separable data. Although the criterion of choosing a proper kernel function without any prior knowledge of the samples is still a data-dependent problem, it is believed that better results can be achieved by combining the kernel function with prior statistical information. Furthermore, physiological information, such as weight, age, gender, etc., can also be considered as input features to improve the classifier performance in future works. 

In addition, Table 2 also shows that the accuracy of the classifier using the fourth-order polynomial kernel is very close to the RBF kernel’s. Therefore, it can be deduced that the order of the five features derived from Equation (4) is possible to be fourth. This deduction is consistent with the known Hagen–Poiseuille Equation, where the flow of incompressible, laminar, steady fluid inside a cylindrical, solid tube is proportional to the fourth order of vessel radius, which is also proportional to PI based on Equation (3). More future works can be conducted on related topics about extracting other features for improving classifiers, and about determining the exact function for assessing AVF by combining numerical analysis and fluid mechanics simulation. 

A comparison table between the current study and other previous research is shown in Table 4, where the proposed PPG sensor renders high accuracy and a low type II error with an adequate number of subjects. Table 4 also shows that though the bilateral PPG sensors using neural networks [5] claimed the highest accuracy, the sensors’ sizes are too large to turn into a portable device. Moreover, the prior research of the authors [6] using a single PPG sensor with the neural network algorithms reveals an extremely high type II error, which may lead to delayed treatments in clinical practice. As for the researches presented in References [2,3], though acoustic sensors using stethoscope auscultation show promising results, the long tubes of the stethoscope sensors are inferior to a PPG sensor for device portability. However, it is possible that a sensor fusion system combining both optical PPG sensors and acoustic stethoscope sensors can be developed in the future to provide ESRD patients a more accurate and reliable solution to AVF assessments. 

There are still some limitations in the proposed PPG sensors, but these can be improved in future works. First, though the proposed PPG sensor can be operated by the users themselves easily without the aid of medical personnel, it is still inferior to other handheld-free devices [2,3,5]. Moreover, despite many signal processing algorithms designed in the system, serious motion artifacts and ambient light interferences may still lead to failure in assessment. Therefore, to overcome the issues of motion artifacts and ambient light interferences, as well as to achieve handheld-free operation of devices, new wearable patch-type PPG sensors for assessing health of AVF are currently under development by the authors’ group, which is supposed to be more prospective then portable-type PPG sensors. The flexible patch-type PPG sensor can be attached tightly to a patients’ skin to work against the ambient light interferences and motion artifacts. These patch-type PPG sensors are expected to be publicized in the near future. 

## 6. Conclusions

In this work, a portable and wireless PPG sensor system for assessing AVF quality using a class-weighted SVM classifier was proposed, aiming at providing a small-sized and inexpensive sesnor module, as opposed to conventional bulky and expensive Doppler machines. Towards the development of the sensor, the readout circuitries and digital signal processing algorithms were designed to increase the SNR of PPG signals. With the sensor module developed, the class-weighted SVM classifiers were used to assess AVF quality. The assessment results were provided to doctors for diagonosis and detemining ensuing proper treatments. Finally, the experimental results by the proposed PPG sensor with a class-weighted SVM classifier using RBF kernel achieved successfully high accuracy (89.11%) and a low type II error (9.59%). 

## Figures and Tables

**Table 1:** Five input features for the support vector machine (SVM) classifier.

Symbol	Measurement	Definition
PI	The Proposed PPG Sensor	Perfusion index defined in Equation (12)
SpO_2_	Oximeter	Blood oxygen saturation levels
SBP	Electronic Sphygmomanometer	Systolic blood pressure
DBP	Electronic Sphygmomanometer	Diastolic blood pressure
*f_HR_*	The Proposed PPG Sensor	Heart rate

**Table 2:** Experimental results of each classifier.

	RBF Kernel	Linear Kernel	2-nd Order Polynomial	3-rd Order Polynomial	4-th Order Polynomial
Kernel Scale (*σ*)	6.1585	-	-	-	-
Misclassification Cost of Positive Class (*C*^+^)	161.8024	1.7159	0.4887	0.1053	0.0603
Misclassification Cost of Negative Class (*C*^−^)	420.6862	4.4615	1.2706	0.2738	0.1567
Accuracy	89.11%	68.32%	75.25%	83.17%	87.13%
Sensitivity	90.41%	68.49%	75.34%	86.30%	90.41%
Type II Error	9.59%	31.51%	24.66%	13.70%	9.59%

The ratio of *C*^+^ / *C^−^* is set to the ratio of positive samples and negative samples.

**Table 3:** Reproducibility test of the proposed PPG sensors.

		RBF Kernel	Linear Kernel	2-nd Order Polynomial	3-rd Order Polynomial	4-th Order Polynomial
Patients #1	Ground Truth	P	P	P	P	P
	First Measurement	P	P	P	P	P
	Second Measurement	P	P	P	P	P
	Third Measurement	P	P	P	P	P
	Accuracy	100%	100%	100%	100%	100%
Patients #2	Ground Truth	N	N	N	N	N
	First Measurement	N	N	N	N	N
	Second Measurement	N	N	N	N	N
	Third Measurement	N	N	N	N	N
	Accuracy	100%	100%	100%	100%	100%
Patients #3	Ground Truth	P	P	P	P	P
	First Measurement	P	N	P	P	P
	Second Measurement	P	N	P	P	P
	Third Measurement	P	N	P	P	P
	Accuracy	100%	0%	100%	100%	100%
Patients #4	Ground Truth	N	N	N	N	N
	First Measurement	N	P	P	P	N
	Second Measurement	N	P	N	N	N
	Third Measurement	N	P	N	N	N
	Accuracy	100%	0%	66.67%	66.67%	100%
Patients #5	Ground Truth	P	P	P	P	P
	First Measurement	P	P	P	P	P
	Second Measurement	P	P	P	P	P
	Third Measurement	P	P	P	P	P
	Accuracy	100%	100%	100%	100%	100%

P denotes the healthy AVF (Positive Class), and N denotes the dysfunctional AVF (Negative Class).

**Table 4:** Comparison table among developed methods of assessing AVF.

	Wu J. X. et al. (2015) [45]	Yeih D. F. et al. (2014) [2]	Wang H. Y. et al. (2014) [3]	Du Y.-C. et al. (2018) [5]	Chiang P. Y. et al. (2017) [6]	This Work
Sensor	Ultrasound	Stethoscope Auscultation	Stethoscope Auscultation	Bilateral PPG	Single PPG	Single PPG
Principle	Doppler	Acoustic	Acoustic	Optical	Optical	Optical
Communication	Wired	Wireless	Wireless	Wired	Wireless	Wireless
Assessing Algorithm	Color Relation Analysis	SVM	Neural Network	Neural Network	Neural Network	SVM
Size	Large	-	9 cm × 4 cm × 2 cm	Large	9 cm × 8 cm × 4 cm	9 cm × 3.5 cm × 1.5 cm
Number of Subjects	50	22	479	11	40	101
Accuracy	83%	84.3%	87.8%	94.82%	R^2^ = 0.7176	89.11%
Type II Error	-	16.7%	10.75%	-	> 50%	9.59%
